# Silk Fibroin-Sheathed Conducting Polymer Wires as Organic Connectors for Biosensors

**DOI:** 10.3390/bios9030103

**Published:** 2019-08-28

**Authors:** Yanke Jiang, Meng Xu, Vamsi K Yadavalli

**Affiliations:** 1 Department of Chemical and Life Science Engineering, Virginia Commonwealth University, 601 W Main Street, Richmond, VA 23284, USA; 2 College of Environment and Resources, Chongqing Technology and Business University, No.19 Xuefu Avenue, Nanan district, Chongqing 40067, China

**Keywords:** conducting fiber, flexible, biosensor, PEDOT:PSS, biodegradable

## Abstract

Conductive polymers, owing to their tunable mechanical and electrochemical properties, are viable candidates to replace metallic components for the development of biosensors and bioelectronics. However, conducting fibers/wires fabricated from these intrinsically conductive and mechanically flexible polymers are typically produced without protective coatings for physiological environments. Providing sheathed conductive fibers/wires can open numerous opportunities for fully organic biodevices. In this work, we report on a facile method to fabricate core-sheath poly(3,4-ethylenedioxythiophene):poly(styrenesulfonate) PEDOT:PSS-silk fibroin conductive wires. The conductive wires are formed through a wet-spinning process, and then coated with an optically transparent, photocrosslinkable silk fibroin sheath for insulation and protection in a facile and scalable process. The sheathed fibers were evaluated for their mechanical and electrical characteristics and overall stability. These wires can serve as flexible connectors to an organic electrode biosensor. The entire, fully organic, biodegradable, and free-standing flexible biosensor demonstrated a high sensitivity and rapid response for the detection of ascorbic acid as a model analyte. The entire system can be proteolytically biodegraded in a few weeks. Such organic systems can therefore provide promising solutions to address challenges in transient devices and environmental sustainability.

## 1. Introduction

Flexible (bio)electronics are an emerging class of technologies with promising applications in areas such as human–machine interfaces, soft robotics, and biosensors [1,2]. To realize their function, such devices require conducting or semiconducting fibers/wires, which are fabricated from intrinsically conductive materials to connect various components and external power sources. While metals are commonly used owing to their high conductivity and low cost, they tend to be easily oxidized under ambient (and especially physiological) conditions, often have poor response to bending or flexion, and can possess a mechanical mismatch in terms of weight and modulus at soft tissue interfaces [3]. With a few exceptions, they are not biodegradable [4]. As an alternative, conducting polymers have been proposed as enabling technologies for organic biosensors and bioelectronics [5,6]. Polymers provide advantages in terms of soft, stretchable, and mechanically conformable properties for interacting with biological systems [7]. They can provide solution processing, biocompatibility, and degradability. Recently, there has been a great deal of interest in engineering fibrous materials to become conductive through blending, coating, or integrating with electrically conductive materials [8,9,10]. In order to use them for flexible or implantable applications, electronic textiles, and skin interfaces, fibers are sought with high performance (conductivity and mechanical properties), while possessing biocompatibility, degradability, and sustainability [11,12].

To date, various conducting polymer fibers have been formed from polyaniline, polypyrrole, and poly (3, 4-ethylenedioxy thiophene): poly (styrenesulfonate) (PEDOT:PSS) [13,14,15]. Specifically, PEDOT:PSS has been widely studied due to its spinnability and easy processability into large-scale industrial fibers [16]. Spun PEDOT:PSS fibers have been shown with tunable electrical conductivity that can be varied from 0.1~4380 S/cm [17,18]. Their conductivity can be enhanced by orders of magnitude by doping or de-doping with various polar solvents, post-heated treatment using UV light, or by changing the spinning solution [19,20,21]. Despite being highly conducting, their mechanical strength tends to be low, and their electrical properties can change under deformation or in physiological environments [22]. Exposed wires are not only susceptible to the external environment, but can affect surrounding tissue [23]. Providing a protective sheath around the fibers can address these concerns. While sheathed metallic wires are common, polymer sheathing of conducting matrices can protect their connections with external power sources or readouts. However, most research on organic conducting wires and fibers has primarily focused on their fabrication, with only a few examples of core-sheathed wires or connection to functional devices shown [15,24,25].

In this work, we report on the fabrication of a core-sheath PEDOT:PSS–silk fibroin wire that is connected to a flexible biosensor for detection in a fully organic design. We are inspired by sheathed multifunctional fibers, which have been studied in tissue engineering (e.g., polycaprolactone/polypyrrole (PCL/PPy) and poly(lactic acid)/polypyrrole (PLA/PPy) core-sheath nanofibers [26]) to improve flexibility and operational life [27]. A facile method is used to fabricate long (m), highly conductive PEDOT:PSS fibers. The fibers are then coated with optically transparent silk fibroin to form a sheathed, insulated, fully organic conducting wire. Silk fibroin has been widely studied as a biodegradable and biocompatible biomaterial that can be easily processed into various forms [28]. Recent applications have shown its use in bioelectronics and optics [29,30]. Previously, composites of PEDOT:PSS with silks have been reported. For instance, an electrochemically combined composite with a silk thread was shown, with high conductivity [31]. Electroconductive silk fibers were shown for use in fabrics and fiber transistors [32]. However, a core–sheath configuration and/or connection to a biosensor was not shown.

This process is conducted using photolithography, which allows connection between various devices using a conducting ink. Specifically, we use the wires as connectors to flexible, fully organic biosensors formed from silk biomaterials [33]. Note that we use the term fibers and wires interchangeably, as the conducting fibers behave as connecting wires in our fully organic system. The function of the biosensor is demonstrated by the detection of a physiologically relevant model analyte (ascorbic acid). The wires allow the formation of a fully organic, potentially degradable, free standing device that can be linked to an external interface (viz. a potentiostat) without the use of metal interconnections. Both single/bundled fibers as well as multiple parallel wires can be fabricated. The conductivity and stability is tested for both bare and sheathed wires. The results indicate that this sheathing process effectively insulates the PEDOT:PSS fibers in the nonconductive direction, but does not change the conductivity along the wire. Importantly, the insulating silk matrix sheath protects the core, thereby prolonging stability and service life. Both the coated conductive fiber and biosensors are light, bendable, and inert under ambient and aqueous conditions, making them suitable for flexible bioelectronics. These devices can be envisioned for implantable applications without electrochemical damage to surrounding tissue. Opportunities for the production of biocompatible, sheathed PEDOT:PSS wires for applications in e-textiles and flexible biosensors and bioelectronic devices can therefore be envisioned.

## 2. Experimental Section

### 2.1. Materials

PEDOT:PSS pellets, lithium chloride (LiCl), lithium bromide (LiBr), 2-(Methacryloyloxy)-ethyl isocyanate (IEM), ethanol, 92% H_2_SO_4_, and the dialysis bags (MWCO 3500 Da) were purchased from Sigma-Aldrich (St. Louis, MO, USA) and used as received. The anhydrous dimethyl sulfoxide (DMSO) and formic acid were obtained from Thermo Fisher Scientific (Waltham, MA, USA). Irgacure 2959 photoinitiator was bought from BASF Corporation (Florham Park, NJ, USA). All reagents were at analytical grade unless stated otherwise.

### 2.2. Preparation of Photocrosslinkable Silk Fibroin Proteins

Fibroin was extracted and dialysis purified from *Bombyx mori* silk cocoons, following the established protocols [34]. To coat the fibers, a photocrosslinkable fibroin (also referred to as fibroin protein photoresist (FPP)) was used. The FPP was synthesized as previously reported by our group [35,36]. Briefly, fibroin was dissolved in dimethyl sulfoxide (DMSO) containing 1 M LiCl to form a 1% (w/v) solution. Photoreactive methacrylate moieties were conjugated at 60 °C for 5 h with continuous N_2_ purge. The modified fibroin was precipitated in excess cold ethanol overnight, washed with cold ethanol/acetone (1:1) solution, and centrifuged at 4 °C (three times). Finally, the product was lyophilized to obtain photoreactive fibroin. A photoreactive sericin (SPP) was prepared in the same fashion and used to form conductive inks for the biosensors.

### 2.3. Preparation of PEDOT:PSS Spinning Solution and Fibers

**Figure 1:**
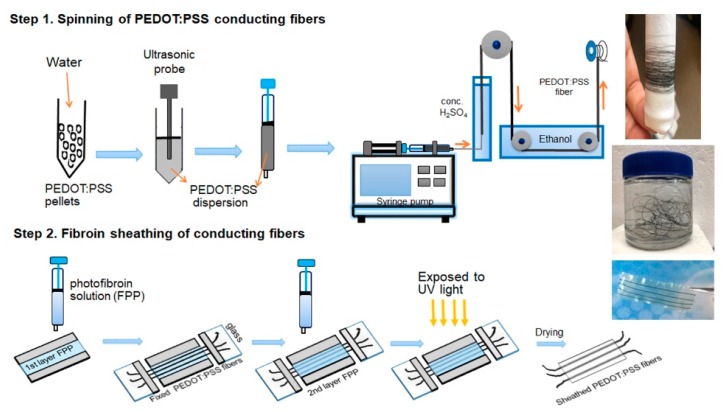
Schematic showing the fabrication of the silk fibroin sheathed conducting PEDOT:PSS fibers. The inset shows the extremely long lengths of fibers that can be easily spooled and collected, as well as the final sheathed (multi) fiber.

PEDOT:PSS pellets were dispersed in water, followed by ultrasonication, to form homogeneous stock solutions of 2.5%, 5%, and 10% (w/w). Fiber spinning was carried out using a modification of a previously reported wet-spinning method [37]. Briefly, the solution of PEDOT:PSS was extruded via a syringe pump (Harvard Apparatus PHD 2000) into a 98% H_2_SO_4_ coagulation bath (Figure 1 Step 1). The extrusion was performed from the bottom of the bath using spinning flow rates of 1.5 to 3 mL/h. Blunt needles of 21-, 27-, and 34-gauge (nominal inner diameters of 514 µm, 210 µm, and 108 µm, respectively) were used as spinnerets to produce fibers with different diameters. The spun fibers were directly washed using an ethanol/water mixture (3:1 v/v) to remove any residual H_2_SO_4_. The PEDOT:PSS fibers were collected on a reel, then dried at room temperature.

### 2.4. Fabrication of Sheathed Single or Multiple PEDOT:PSS Fibers

Sheathed PEDOT:PSS fibers were formed by sheathing the conductive fibers in the photofibroin via photocrosslinking at an ambient environment. The solution used formic acid as the solvent and consisted of 13.2% (w/v) of FPP with 4.4% of Irgacure 2959 photoinitiator. The photoreactive, optically transparent coating solution was applied on a glass microchannel support to form the bottom thin layer of film for fiber coating (Figure 1 Step 2). The PEDOT:PSS fibers were then straightened and anchored on the bottom. The solution was then drop-cast onto the bottom film. Following the evaporation of the excess solvent, the sheathed fiber was crosslinked using UV light for 5 s (OmniCure S1000 UV Spot Curing lamp (Lumen Dynamics, Ontario, Canada) equipped with a 320–500 nm filter). Exposure of 365 nm (2 mW cm^−2^) was used. Free-standing, sheathed, conducting fibers could then be obtained by separating from the glass support using water immersion.

### 2.5. Electrical Characterization of the Sheathed PEDOT:PSS Wires

The electrical characterization and electrochemical measurements were conducted using a Gamry Interface 1010E Potentiostat (Gamry Instruments, Warminster, PA, USA). The electrical conductivity of the uncoated PEDOT:PSS fibers and coated wires were characterized by cyclic voltammetry (CV) using a two-electrode configuration. To test the success of the coating, Cu tape was attached to the exposed portion of the fiber at different positions on the sheath. The ability of the silk fibroin to form an electrically insulating sheath on the conductive fibers was tested. CV was conducted in a scanning range of −1 to 1 V at a scan rate of 100 mV/s. The fibers were fixed on a glass support using Cu tapes and connected to the potentiostat (Figure S1 in the Supplementary Materials). The electrical stability of the uncoated fibers was also tested using the same setup. The PEDOT:PSS fibers were cut into 3-inch pieces and immersed in water for 0–28 days. At seven-day intervals, fibers (three replicates/experiment) were taken out, and their conductivity tested in dry condition.

### 2.6. Fabrication of a Flexible Biosensor with the Integration of Coated PEDOT:PSS Fibers

To form integrated biosensors, the sheathed PEDOT:PSS wires obtained above were attached to flexible electrodes. Flexible organic electrodes were formed in a two-step process, as earlier reported [33,38]. A biocomposite conducting ink (2.5% (w/v) of SPP, 28% (w/v) PEDOT:PSS, and 0.5% (v/v) of Darocur 1173 photoinitiator in water) was used to form the micropatterned biosensor. The electrodes were formed on thin films (7.5% (w/v) in formic acid) formed by drop-casting on a glass slide, followed by photolithography to form micropatterned electrodes. To fabricate the entire biosensor devices, the sheathed PEDOT:PSS wires were connected to the flexible electrodes by applying the biocomposite conductive ink prepared above. The ink was drop-cast at the junction of the sheathed wires and the flexible electrodes. After the solvent was completely evaporated under darkness in a fume hood, the junction was photocrosslinked using UV light to form a conductive connection. Finally, the junction was sealed using photofibroin solution (7.5% (w/v) in HFIP) and exposure under UV light for 1.5 s. The entire biosensor could be detached from the glass support by immersing in water and peeling off, resulting in a free-standing device.

### 2.7. Electrochemical Detection of Ascorbic Acid

The integrated biosensors (organic electrodes + sheathed wires) were tested for their performance as biosensors by using the electroactive ascorbic acid as a model analyte. A standard three-electrode setup was used, with the fabricated flexible devices as the working electrode, Ag/AgCl as reference electrode, and platinum as counter electrode. A chronoamperometric mode (with constant potential of 0.4 V) was applied to monitor the response of the biosensor to the continuous addition of ascorbic acid in phosphate buffered saline (PBS) buffer (0.1 M, pH 7.4).

## 3. Results and Discussion

While various forms of organic conducting fibers including the use of PEDOT:PSS either by itself [17,18] or with silk [39] have been shown, the focus has been on the processing techniques to enable continuous processing and homogeneous fibers with good conductivity and structure. However, a few aspects have not been well studied to date. Primarily, these fibers are typically not sheathed or insulated, which can compromise their stability. Despite being highly conducting, the mechanical strength of pure PEDOT:PSS fibers tends to be low, and susceptible to changes in electrical properties under deformation or in physiological environments [22]. Furthermore, unsheathed wires have the disadvantage of potentially causing electrochemical damage to surrounding tissues [23]. Finally, their ability to be connected to devices such as biosensors has not been shown. This works shows the fabrication of highly stable, core-sheath PEDOT:PSS–silk fibroin wires that can connect to a flexible biosensor for detection in a fully organic configuration.

### 3.1. Wet Spinning of Pure PEDOT:PSS Fibers

In order to obtain reproducible results and prevent extraneous factors, PEDOT:PSS pellets were directly dissolved into deionized water used as spinning dope in the experiments reported herein. Aqueous dispersions with 2.5 wt.%, 5.0 wt.%, and 10 wt.% PEDOT:PSS were investigated to spin conductive fibers. No doping/de-doping agents were added to the spinning solution and no hot-drawing process was used. The spinning parameters were initially optimized to achieve the continuous production of PEDOT:PSS fibers with uniform conductivity [29]. An H_2_SO_4_ solution of 92% was used as the coagulation bath and PEDOT:PSS dispersion was injected from the bottom of the bath (Figure 1). This is because the density of PEDOT:PSS is lower than H_2_SO_4_ solution. Blunt needles (21-, 27-, and 34-gauge blunt needles with nominal inner diameters of 514 µm, 210 µm, and 108 µm, respectively) were used to produce fibers of different diameters (Table 1, additional images are shown in Figure S2 in the Supporting Information). The as-spun PEDOT:PSS fibers remained in the H_2_SO_4_ solution for 10 min. Following this, excess H_2_SO_4_ was washed off using an ethanol/water (3:1) mixture, and the fibers dried at room temperature. The spun fiber could be directly wound on a spool with a well-distributed diameter, as shown in Figure 1. This process results in extremely long and uniform fibers, with smooth surfaces and circular cross-sections (Figure S3). Single continuously drawn conducting fiber over several meters in length could be easily produced.

### 3.2. Properties of PEDOT:PSS Conducting Fibers

Both the concentration of the starting PEDOT:PSS solution as well as the gauge size of the needles can affect the spinnability and the conductivity of the fibers Table 1. Shorter fibers were cut at random from the reels and fixed on a glass slide in order to test their morphology and conductivity (Figure S1). The wire diameters were obtained at five random points along the fiber (Figure S2). Expectedly, the fiber diameter increased with a decrease in needle gauge, corresponding to a higher inner diameter. However, the fiber diameter is also a function of the concentration of the PEDOT:PSS, and thereby the viscosity of the solution. Using 32 G needles with a nominal inner diameter ~100 µm and a 5 wt.% solution, we obtained the thinnest wire diameter of ~12 μm. In contrast, the diameter for 2.5 wt.% was ~20 µm. At 10 wt.% concentration of PEDOT:PSS, the solution was too dense to be extruded through this gauge. When using thicker 21 G needles (nominal inner diameter ~500 µm), the diameter of the spun fiber was about 1.6 times higher. In general, the wire diameter was much lower than the spinneret diameter, which is expected due to the loss of the water as the solution is extruded into the concentrated H_2_SO_4_. The acid not only removes the water, but also improves the overall conductivity [17]. Indeed, it has been reported that the concentrated sulfuric acid removes the PSS component, rendering the fibers insoluble in water [40].

**Figure 2:**
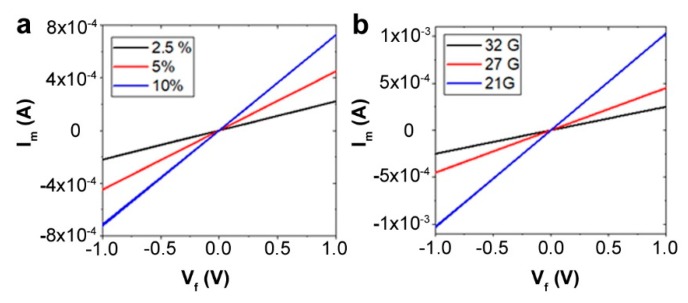
(**a**) Current-Voltage (I–V) curves of PEDOT:PSS fibers spun using different concentrations of PEDOT:PSS. At higher concentrations, the resistance is lower. (**b**) I–V behavior as a function of the needle diameter for the same concentration of PEDOT:PSS (5%).

The resistance of the fibers decreased with an increase in PEDOT:PSS concentration (Table 1). It may be noted that the conductivity is a function of both the resistance and the fiber diameter. The conductivity σ of the spun fibers and *I_m_/V_f_* were measured and calculated from the diameter and cyclic voltammetry curves, as shown in Figure 2. The conductivity can be calculated using the following equation: σ = (I*L)/(V*πr^2^*)*, where *I* = current (A); *L* = length (m); *V* = voltage (V), and *r* = radius (m) of the wire. The fabricated PEDOT:PSS fibers showed extremely competitive conductivity, in line with previous reports [17,37]. At 5 wt.%, the thinnest wires showed a very high conductivity of over 700 S/cm. The performance metrics at 2.5% were high, but the fibers were thinner, owing to the low viscosity of the dope solution. Therefore, optimizing for the processing conditions, the fiber diameter (strength), and conductivity, we selected fibers with 10 wt.% PEDOT:PSS, which were extruded using a 27 G needle at 0.25 µL/min. This composition resulted in highly stable fibers ~50 µm diameter that can be easily handled, while showing a conductivity of ~140 S/cm.

### 3.3. The Stability of PEDOT:PSS Fibers in Water

**Figure 3:**
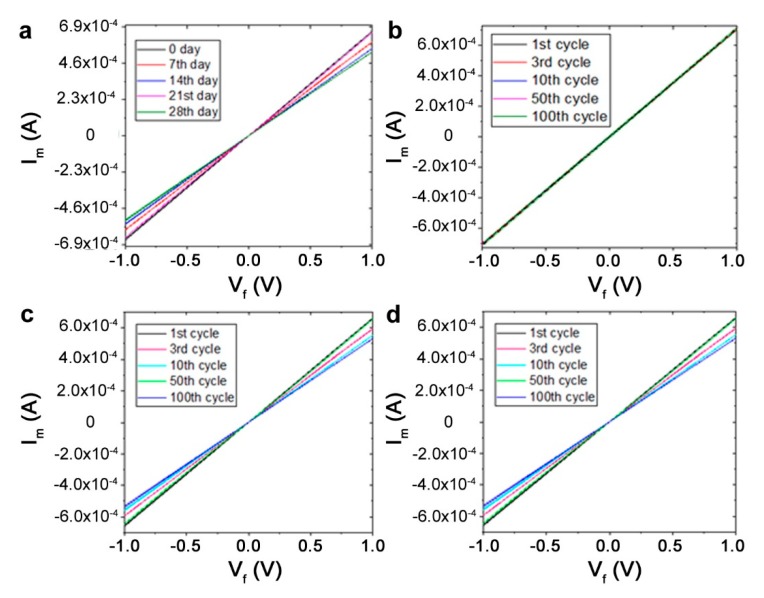
Conductivity and cyclic stability for the conducting PEDOT:PSS wires. (**a**) I–V curves obtained from fibers immersed in water over one month. The minor deviations show the high stability of the fibers. (**b**) Average value of conductivity over 100 cycles of testing. (**c**) Cyclic testing after one week of immersion. (**d**). Testing after four weeks of immersion.

The optimized fibers were used to explore the stability of the fibers formed for various applications. The wires were immersed in water for one month, with samples extracted each week (seven-day intervals). Three samples were extracted at these time points and the diameters and conductivity were measured as discussed above (Table 2, Figure 3). Importantly, our results show the high stability of the wires formed, with no obvious change in diameter even after one month. The conductivity of the original fiber only slightly changed from 144 S/cm to 133 S/cm after one day and remained unchanged over 100 cycles of testing. Remarkably, the conductivity dropped by only ~10% after two weeks and ~20% after one month. The wires were stable over 100 cycles of testing, with only a ~6% variation after one week and ~15% variation even after one month (Figure 3). Critically, the wires maintained their function for conduction with competitive metrics, and could therefore be used to provide power for devices. Given that we were interested in designing biodegradable sensors and devices that can break down over after a short functional period on the order of weeks or months, this performance can be considered satisfactory.

### 3.4. Sheathing of Conductive PEDOT:PSS Fibers to Form Insulated Organic Wires

**Figure 4:**
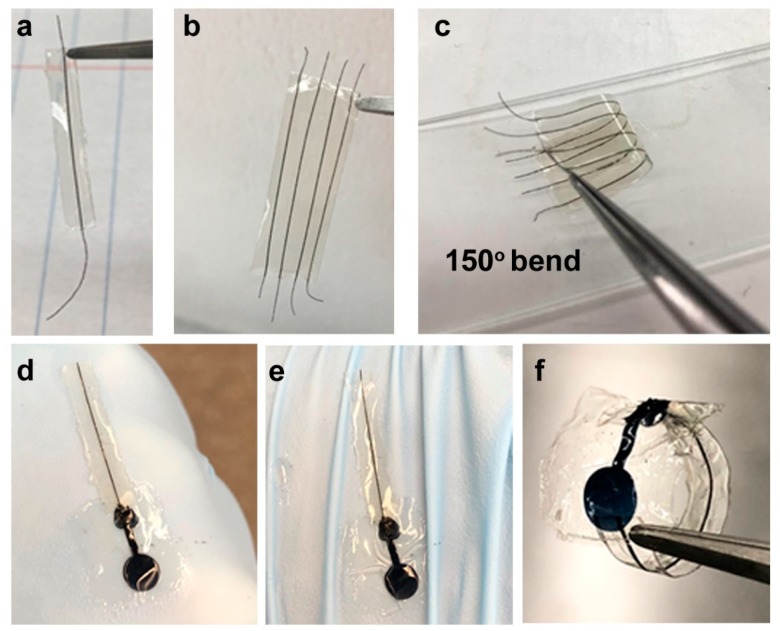
(**a**) Single and (**b**) multiple fibers can be sheathed using this technique, allowing for potentially multiplexed biosensors. (**c**) The PEDOT:PSS fibers are stable to bending in the sheaths. (**d**) Integrated wire and flexible biosensor. The microfabricated electrode biosensor is formed from an organic conducting ink and is connected to the wires to form a functional device. (**e**) The mechanically flexible device can conform to irregular surfaces. (**f**) Bending without breakage.

One of the objectives of this research was to form a continuous process for the formation of core–sheath organic wires. Here, the insulation was provided by coating the wires with the intrinsically non-conducting silk protein fibroin. Specifically, the photofibroin (FPP) provided an avenue to form a water-insoluble insulation for the wires, with the liquid to solid transition provided by UV crosslinking. Initially, a dip coating method was explored to coat the PEDOT:PSS fibers using the photofibroin solution. However, this was not amenable to continuous processing and the fiber sheaths were not uniform. Subsequently, a method of suspending the wires in the solution was developed. A series of photofibroin solutions in formic acid (4% to 15% wt.) were studied. A 13.3 wt.% concentration was chosen as the optimal solution because of its similar density to the spun PEDOT:PSS fibers. This allowed the fibers to maintain their positions in the solution without rising or sinking in the absence of an external force. The solutions had a high surface tension and low fluidity, which allowed a direct coating procedure on the fibers. In order to conserve material, a quasi-microfluidic flow channel was formed and the fibers were suspended in the middle. By controlling the depth of the channel, the coating thickness could be controlled. The use of the microfluidic channel allowed the rapid formation of sheathed wires of several inches in length. After drying the coating solution and crosslinking in UV, the films sealing the PEDOT:PSS fibers could be directly peeled off from the glass (Figure 4a). Either single or multiple fibers could be coated using this method (Figure 4b). The use of multiple fibers coupled with micropatterned electrodes, as we show, can result in multiplexed biosensors on the same platform. Most interestingly, the coatings were optically transparent and flexible, providing a protective sheath for the conducting fibers and improving their overall strength. The wires could be bent even over 150° ^(^Figure 4c, right) without breaking.

**Figure 5:**
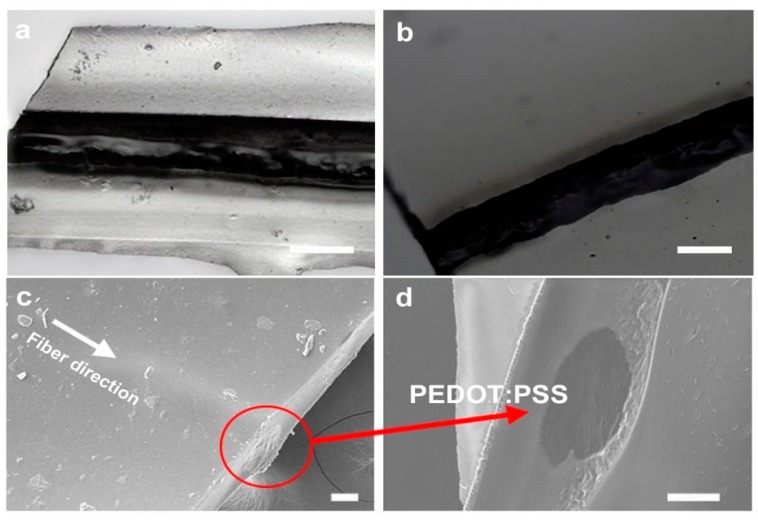
(**a**,**b**) Optical imaging of upper and lower surfaces, showing the sheathed dark conducting PEDOT:PSS wire enclosed in the transparent fibroin sheath. (**c**) SEM imaging of the sheathed wires. (**d**) Cross-section across the wire showing the dark fiber surrounded by the insulating silk matrix. Scale bar = 50 µm in all panels.

**Figure 6:**
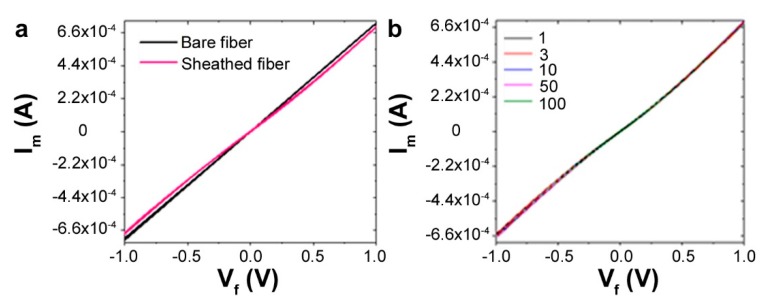
(**a**) The conductivity comparison (I–V) between the bare and sheathed fibers. (**b**) Conductivity across multiple cycles of testing for the sheathed fibers reveals their stability.

The surface and the cross section of the sheathed fibers were observed by optical microscopy and scanning electron microscopy (SEM). The micrographs show uniform surfaces and a well-defined morphology of the wires and the sheath. The PEDOT:PSS fibers were tightly wrapped in the middle of the insulating fibroin sheath Figure 5. The presence of the silk fibroin insulating matrix around the conducting fibers formed a core–sheath configuration, with the conducting PEDOT:PSS wire well surrounded by the silk sheath. In order to verify that the sheath was performing as an insulator without affecting the fibers themselves, the conductivity along the axis of the PEDOT:PSS fiber was tested. The *I_m_/V_f_* curve of the sheathed PEDOT:PSS fibers was nearly similar to that of the bare fibers (Figure 6a). The conductivity of the sheathed fiber was about 138 S/cm, close to the value of the bare fibers (144 S/cm), showing that the insulation protocol did not affect the wires. The *I_m_/V_f_* value maintained its stability over 100 cycles (Figure 6b). Next, the silk coating itself was tested to verify that there was no leakage or cross-conductivity through the sheath. No signal could be detected across the wire. Cyclic voltammetry was also used across the insulated wires and only nA levels of current were detected, which are close to noise (Figure S5). Therefore, we can conclude that the PEDOT:PSS fibers were successfully insulated by the silk fibroin sheath, which provided electrochemical and physical isolation to the external microenvironment.

### 3.5. Detection of Ascorbic Acid Using the Assembled Biodegradable Biosensor

Typically, even flexible and biodegradable biosensors use conventional metallic wires to form interconnections. In our previous work, we showed a flexible, organic biosensor fabricated using a composite of silk sericin and PEDOT:PSS. In this study, for the first time, we showed that the organic, sheathed conducting wires can directly connect with this flexible organic biosensor. The wires therefore allow the interfacing of a device over a long (several cm) distance without the need for any metallic wires. The integrated flexible biosensor was tested for its function. This demonstrated that electrical signals can be sent and received over these wires, resulting in a fully organic biodevice. Ascorbic acid, also known as vitamin C, was used as a model analyte in these experiments. As an electroactive species, it is also a vital component in tissue repair, wound healing, and immune functions [41,42].

**Figure 7:**
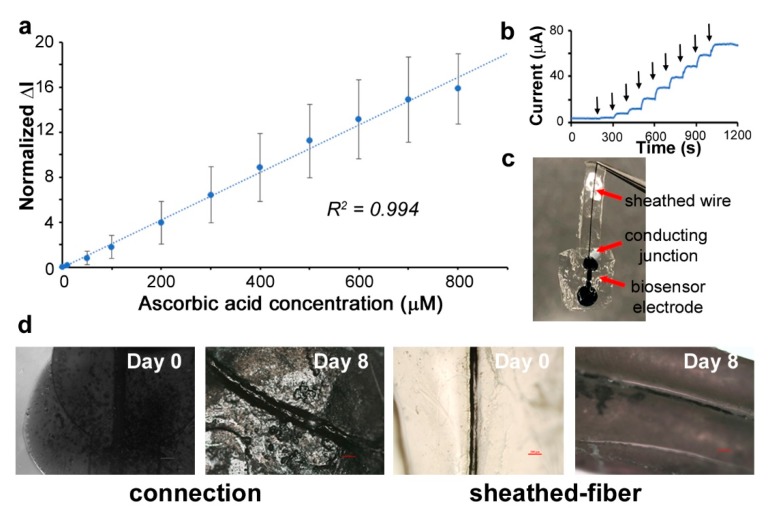
(**a**) Calibration curve for the addition of ascorbic acid. The normalized current response versus the addition of ascorbic acid is shown (three different sensors were tested and the average shown). (**b**) Chronoamperometric addition of ascorbic acid over time (added every 100 s) corresponding to the curve in (**a**). (**c**) Flexible biosensor with the micropatterned organic electrode connected to the core–sheath PEDOT:PSS wire. (**d**) The entire organic device may be controllably degraded. Optical imaging showing the progression of degradation of the connection and wire under proteolytic action.

The assembled biosensor was constructed, with the micropatterned organic electrode connected to a sheathed PEDOT:PSS wire (Figure 7c), resulting in a flexible device. The devices themselves could be bent or conform to irregular surfaces (Figure 4e,f). As seen in Figure 7a, the performance of the biosensor for the detection of ascorbic acid was very effective. The response of the biosensor is shown as the normalized changes of current based on the baseline signal, which is the reading in pure PBS with no ascorbic acid (Δ*I*_nor_ = (*I*_AA_ − *I*_PBS_)/*I*_PBS_). A linear calibration curve was obtained. The inset Figure 7b shows the chronoamperometric response of the biosensor and exhibits that the biosensor had a rapid response time to the addition of ascorbic acid, on the order of a few seconds. The limit of detection (LOD) of the developed biosensor was 1.14 µM, with a very broad range of detection from 0 to 800 µM. This LOD is comparable to what we and others have obtained in previous works using metallic wires to connect the flexible electrodes [33,43]. In an additional experiment, we used this system for the detection of the neurotransmitter dopamine. An effective linear calibration curve was obtained (Figure S6). Thus, these biosensor devices can be used for a range of analytes by simply modulating the electrochemistry. Since the entire device (electrodes and wires) is made of biocompatible and biodegradable materials, they can be potentially envisioned for tissue interfacing applications.

It is known that silk proteins can be proteolytically degraded. These devices were immersed in protease enzyme over several days to observe their degradation and bioresorbability (Figure 7d). A total of 1 mg/mL protease solution at 37 °C was used, which corresponds to a 3× higher concentration than that physiologically available. Following a period of eight days, the connection between the electrode and the wire began to be degraded. This shows that even under the most challenging environments, our devices can function well over one week before failure. This time period can be controlled by controlling the device thickness and insulation layers. Following this, the device can be potentially left behind without the need for explantation, prior to complete degradation, in the form of a transient system [44]. It is important to mention that PEDOT:PSS is itself not biodegradable. Our degradation tests therefore indicate the need to optimize the overall device based on the desired work duration. This would avoid possible electrical failure due to the exposed conductive surfaces. Notably, we used an organic ink as the conducting paste to make the connection between the wire and the biosensor. An insulating organic sheath was used to protect the connection. This implies that our wires can be connected to any biosensor or device to function. Most importantly, the presence of an insulating sheath on the conducting fibers not only provides protection for the wires in a physiological environment, but allows for a tissue interfacing without any resulting electrochemical damage to the surroundings when connected as a biosensor.

## 4. Conclusions

In summary, here we have shown a fully organic, integrated biosensor device fabricated using a conducting ink formed from the polymer PEDOT:PSS dispersed in a silk protein matrix. In order to form connections for the device, we showed the fabrication of conducting PEDOT:PSS fibers/wires using a facile spinning procedure. The fibers can be formed with great fidelity and length, with excellent conducting properties. Most importantly, these fibers can be sheathed in an insulating silk fibroin layer to form conducting wires. The wires can be connected to the micropatterned biosensors to form a fully organic device that is highly sensitive as a biosensor, yet mechanically flexible and degradable. Other than the connection to the potentiostat, no metals were used in this device. Such systems can therefore be explored for a variety of environmental and physiological applications in which the entire device can be programmed to degrade at the conclusion of its functional life.

## Figures and Tables

**Table 1:** Diameter measurement and conductivity of fibers spun from PEDOT:PSS aqueous solution (2.5%, 5%, 10%) using different gauge needles (21 G, 27 G, 32 G with nominal inner diameters of 514 µm, 210 µm, and 108 µm, respectively). The average values from three different fibers are reported.

PEDOT:PSS Concentration	Gauge	Avg. Diameter (μm)	I_m_/V_f_ (A/V)	Conductivity (S/cm)
2.5%	32 G	22.34	2.21 × 10^−4^	214
	27 G	23.60	2. 49 × 10^−4^	216
	21 G	38.36	6.87 × 10^−4^	226
5%	32G	11.53	2.49 × 10^−4^	716
	27G	18.82	4.48 × 10^−4^	483
	21G	29.79	1.02 × 10^−3^	439
10%	27G	49.10	7.13 × 10^−4^	144

**Table 2:** Conductivity test for PEDOT:PSS fibers immersed in water for 1, 7, 14, 21, and 28 days (data reported here are for 3.8 cm fibers extruded using a 27 G needle). The average value from three different fibers is reported. The starting conductivity is 144 S/cm.

Day	Avg. Diameter (μm)	I_m_/V_f_ (A/V) (One Cycle)	Conductivity (One Cycle) (S/cm)	I_m_/V_f_ (A/V) (2–100 Cycles)	Conductivity Stability (2–100 Cycles) (S/cm)
1	48.79	6.57 × 10^−4^	133	6.57 × 10^−4^	133
7	49.18	6.48 × 10^−4^	130	6.85 × 10^−4^–5.40 × 10^−4^	137–128
14	48.81	5.93 × 10^−4^	120	6.35 × 10^−4^–5.53 × 10^−4^	128–112
21	49.12	5.54 × 10^−4^	111	5.96 × 10^−4^–5.06 × 10^−4^	119–101
28	48.91	5.36 × 10^−4^	108	5.68 × 10^−4^–4.73 × 10^−4^	114–95

## Supplementary Materials

The following are available online at https://www.mdpi.com/2079-6374/9/3/103/s1, “Figure S1: Testing procedure to obtain the conductivity for bare and sheathed conducting fibers, Figure S2: PEDOT:PSS fibers spun using different gauge needles, Figure S3: PEDOT:PSS fibers spun using different weight % of the dope solution with the same gauge needle, Figure S4: Optical images showing the fibers and the cross-section SEM images, Figure S5: Testing the insulation efficacy of the silk fibroin sheath, Figure S6: Response of the fully integration biosensor device to additions of the neurotransmitter dopamine.”
