# Supplementary material for: Silk Fibroin-Sheathed Conducting Polymer Wires as Organic Connectors for Biosensors
Source: Biosensors (Basel). 2019 Aug 28;9(3):103. doi: 10.3390/bios9030103 (PMC6784353; doi:10.3390/bios9030103)
Supplement: Supplementary file 1 [file biosensors-09-00103-s001.pdf]

Supplementary Materials

Silk Fibroin-Sheathed Conducting Polymer Wires as Organic Connectors for Biosensors

Yanke Jiang [1,2], Meng Xu [1] and Vamsi K Yadavalli [1,*]

[1] Department of Chemical and Life Science Engineering, Virginia Commonwealth University, 601 W Main Street, Richmond, VA 23284, USA

[2] College of Environment and Resources; Chongqing Technology and Business University, No.19 Xuefu Avenue, Nanan district, Chongqing 40067, China

* Correspondence: vyadavalli@vcu.edu

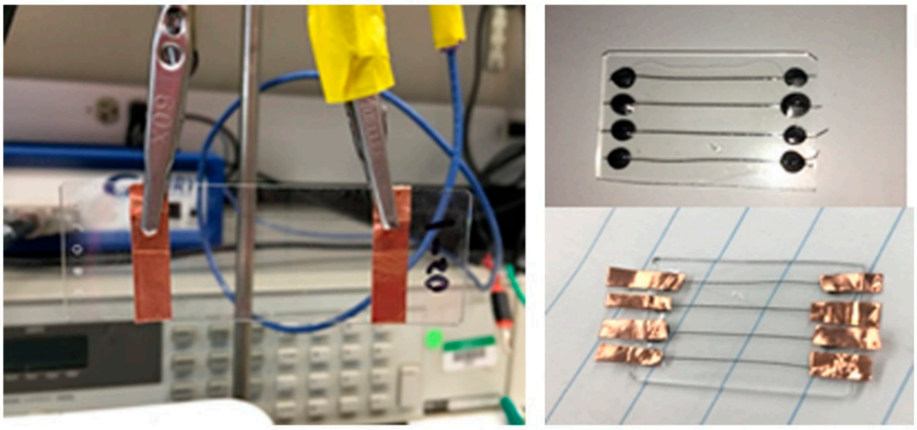

Figure S1. Testing procedure to obtain the conductivity for bare and sheathed conducting fibers.

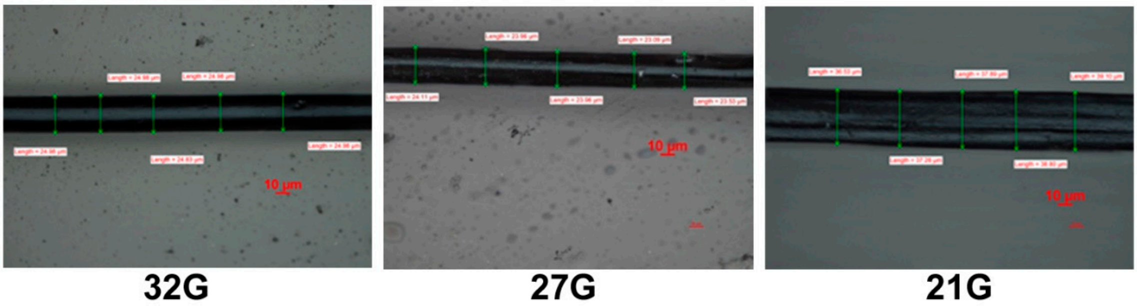

Figure S2. PEDOT:PSS fibers spun using different gauge needles (nominal inner diameters of 21G = 514 μm, 27G = 210 μm and 32G = 108 μm).

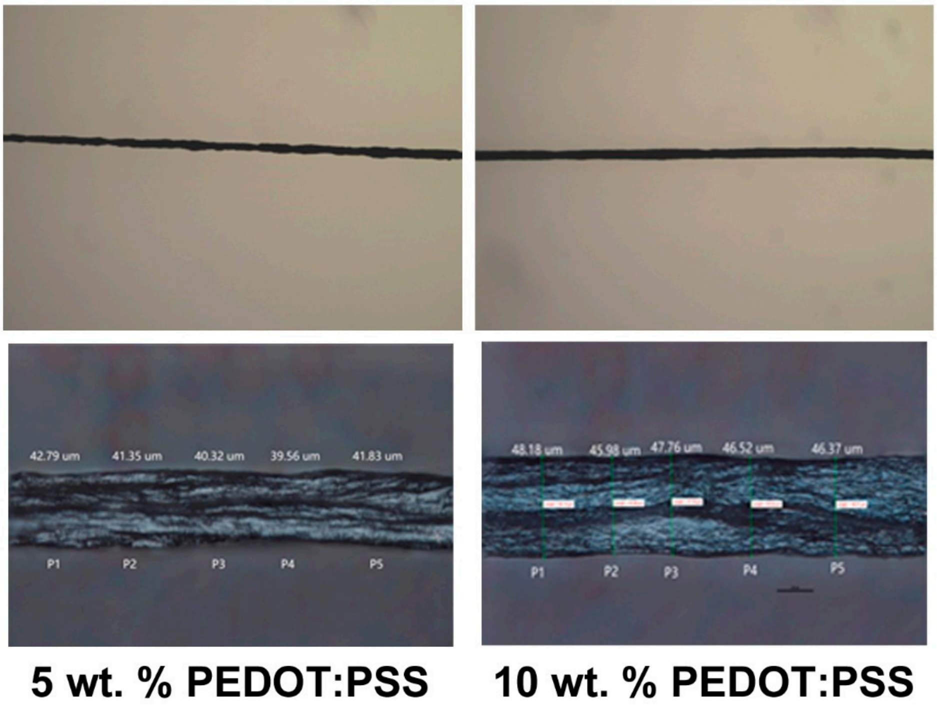

Figure S3. PEDOT:PSS fibers spun using different weight % of the dope solution with the same gauge needle.

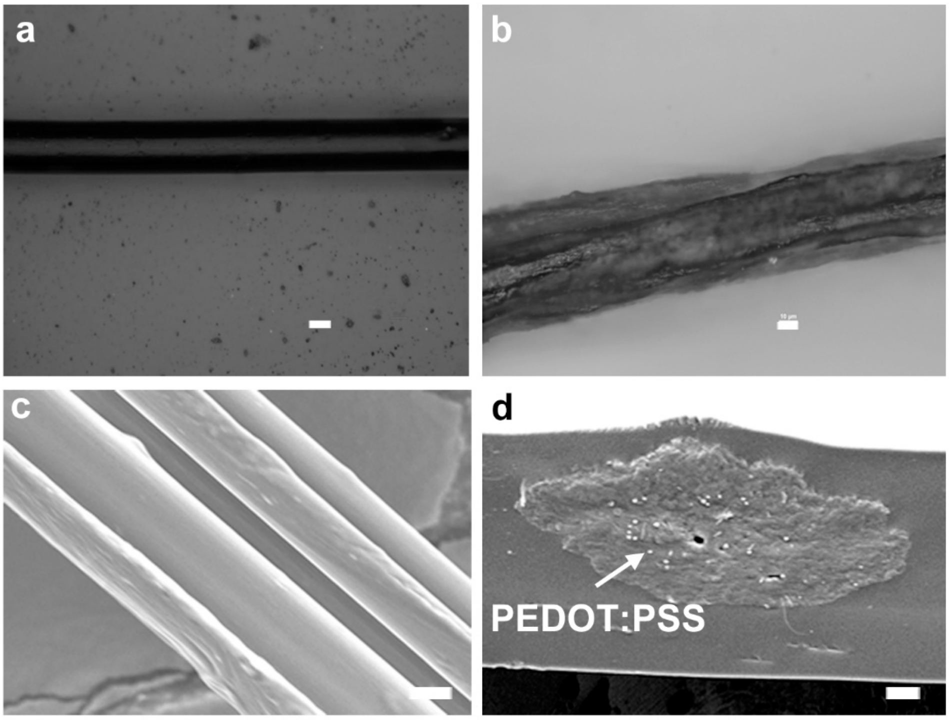

Figure S4. a, **b**. Optical images showing the fibers and the cross-section. **c**, **d** SEM images. Scale bar = 10 µm on all panels.

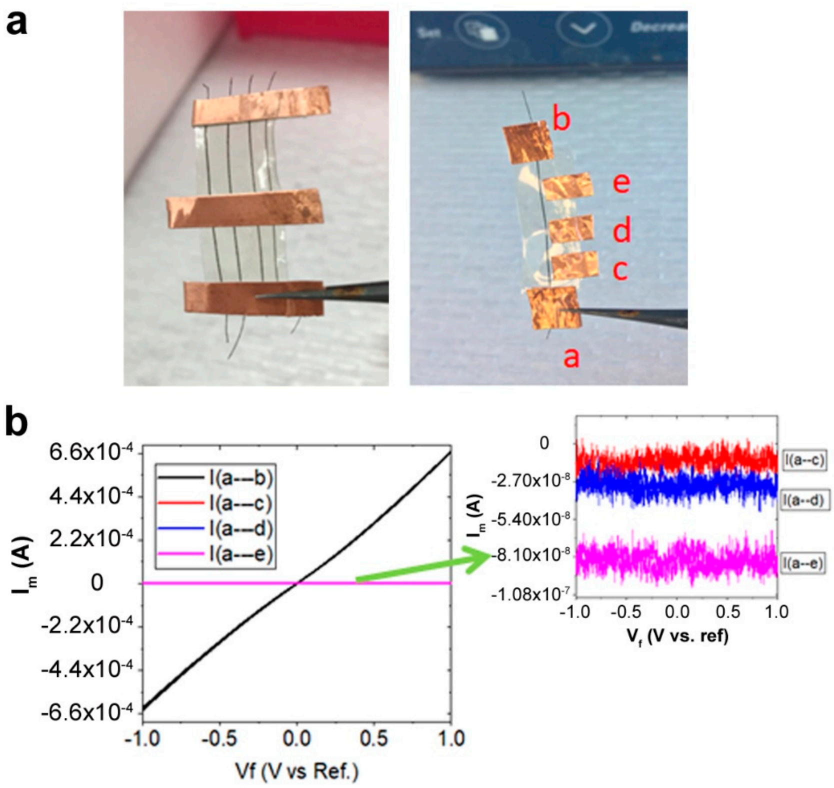

Figure S5. Testing the insulation efficacy of the silk fibroin sheath. (**a**), The conductivity was tested along the sheathed fiber at different points and also across different points. For instance, **a**–**b** measures the conductivity along the fiber, whereas **a**–**c** measures the conductivity from the fiber to the insulating surface. Conductive properties are maintained along the fiber, while the surface itself does not show any conductivity or leakage.

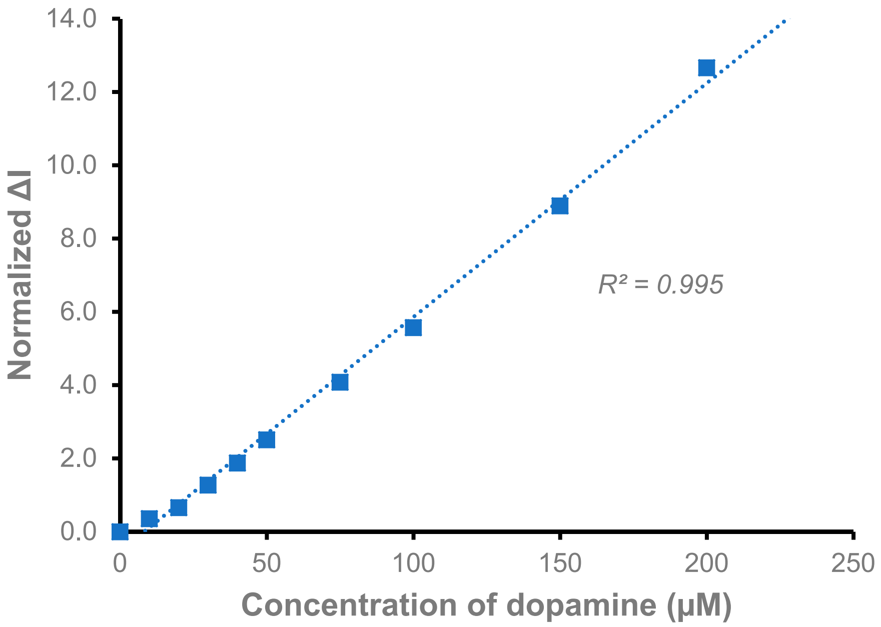

Figure S6. Response of the fully integration biosensor device to additions of the neurotransmitter dopamine. A chronoamperometric setup was used to detect the analyte within the physiological range, showing a linear calibration curve.
